# Misleading Chest Pain: Acute Coronary Syndrome or Lung Adenocarcinoma With Malignant Pericardial Effusion

**DOI:** 10.7759/cureus.65624

**Published:** 2024-07-29

**Authors:** Kajananan Sivagurunathan, Nalayini Jegathesan, Peranantharajah Thampipillai

**Affiliations:** 1 Internal Medicine, Teaching Hospital Jaffna, Jaffna, LKA

**Keywords:** lung malignancy, acute coronary syndrome, malignant pericardial effusion, lung adenocarcinoma, chest pain

## Abstract

Chest pain is the most common presentation of acute coronary syndrome (ACS), but noncardiac causes should be considered when symptoms persist despite treatment or when other clinical features suggest an alternative diagnosis. We report a case of a 60-year-old woman with dyslipidemia who presented with chest pain, exertional dyspnea, and mild dry cough. Initial evaluations, including electrocardiogram and elevated troponin I levels, suggested a diagnosis of ACS. However, her symptoms did not settle with the initial treatment for ACS. Further investigations revealed moderate to massive pericardial effusion and cytology indicative of malignant cells. CT imaging showed a mass in the right lower lobe of the lung with associated bronchial obstruction, lung collapse, and sclerotic bone metastases. Bronchoscopy and biopsy confirmed the diagnosis of invasive adenocarcinoma of the lung. This case emphasizes the essential of considering a broad differential diagnosis, the importance of comprehensive diagnostic workup in patients with persistent chest pain, and stresses the role of interdisciplinary approaches in difficult clinical scenarios.

## Introduction

Chest pain is one of the most frequent presentations at emergency department admissions, demanding prompt evaluation to exclude acute coronary syndrome (ACS), which needs immediate intervention and management. However, chest pain does not always indicate ACS. Still, it also has a spectrum of differential diagnoses requiring a systematic assessment to identify various etiologies that may mimic or coexist with cardiac conditions. This broader differential diagnosis includes pericardial, pulmonary, upper gastrointestinal, musculoskeletal, and psychological causes, each requiring specific diagnostic approaches to ensure accurate diagnosis and appropriate therapeutic measures.

Lung malignancy usually presents with chronic cough, hemoptysis, nonspecific chest pain, and symptoms of metastatic disease. Around one-third of the malignant pericardial effusion is due to lung malignancy [1]. Metastasis to pericardial tissue may be explained by direct mediastinal and hematogenous spread [2].

We present a case of a 60-year-old woman with dyslipidemia who was admitted with chest pain, exertional dyspnea, and dry cough. As she failed to improve with initial management and had other atypical clinical features, it warranted further investigation excluding other causes. The extensive evaluation revealed that she had malignant pericardial effusion due to metastatic adenocarcinoma of the lung.

## Case presentation

A 60-year-old woman with dyslipidemia presented with a two-month history of intermittent chest pain which worsened over the last few days, associated with exertional dyspnea and dry cough. She never smoked. At the time of admission to the emergency department, her vital signs were as follows: blood pressure of 146/89 mmHg, pulse rate of 98 bpm, respiratory rate of 20 cycles per minute, and oxygen saturation of 97%. On auscultation, she had reduced breath sounds in the right lower zone without any added sounds, and her heart sounds were audible without murmurs.

**Figure 1:**
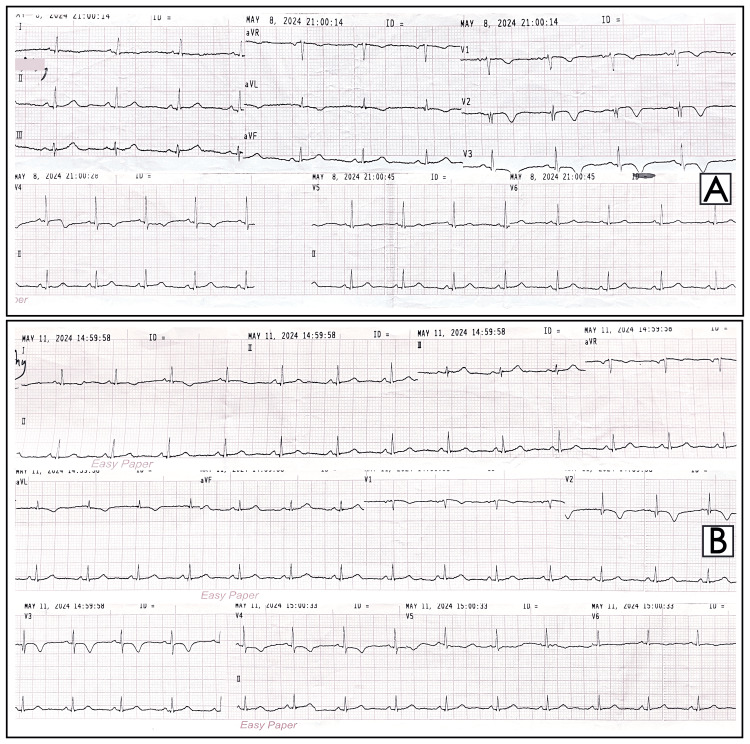
Electrocardiogram (ECG) shows low QRS voltage and T-wave inversions in leads aVL and V1 to V4 (A), with no significant progression on subsequent ECG (B).

The electrocardiogram (ECG) showed low QRS voltage and minor T-wave inversions in leads aVL and V1 to V4, with no progression on subsequent ECGs (Figure 1). Troponin I levels were slightly elevated at 0.124 ng/mL (reference range: <0.120 ng/mL). Given the worsening of her chest pain, ECG changes, and elevated troponin I, she was treated for ACS with antiplatelet therapy and heparin.

**Figure 2:**
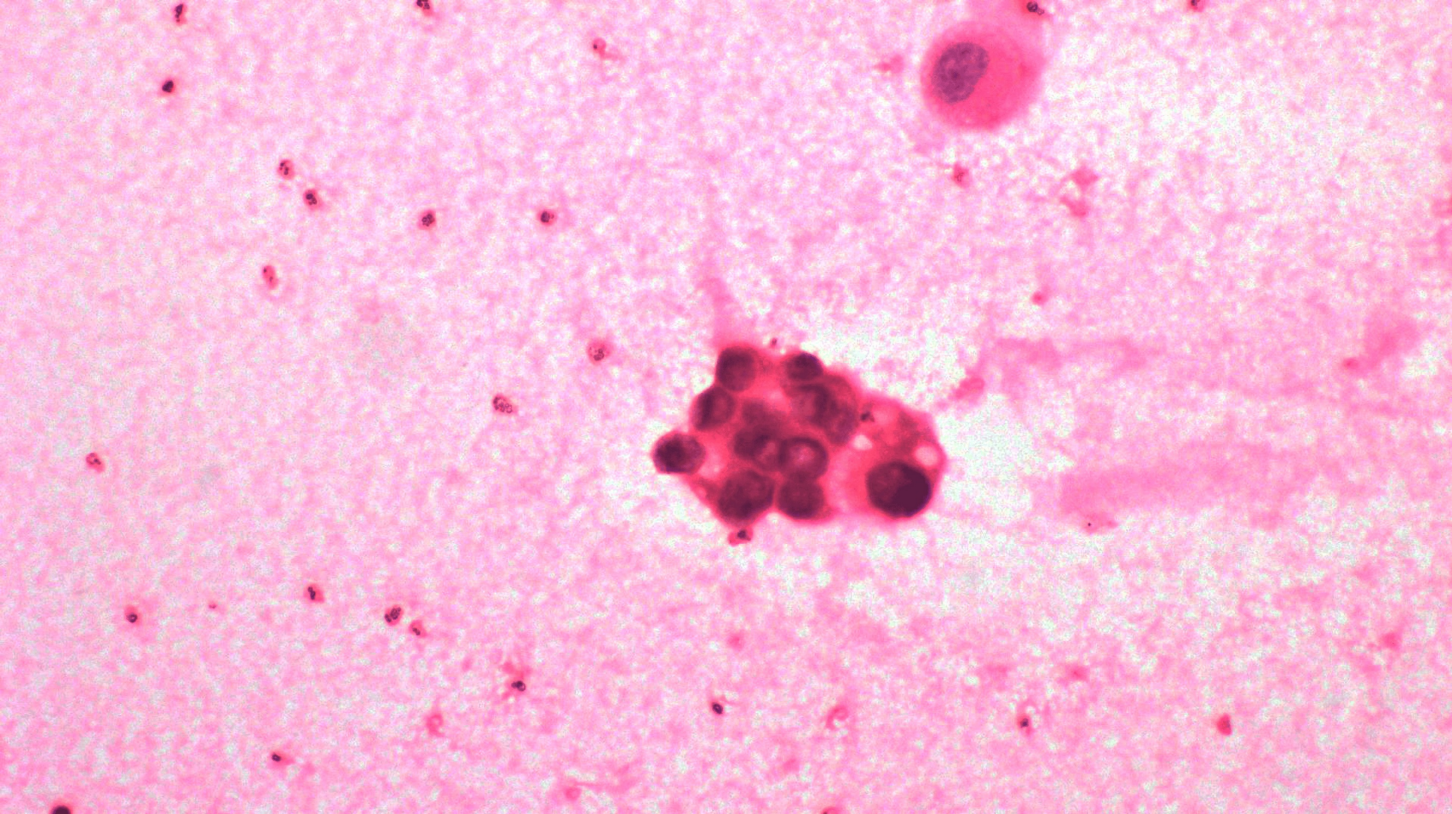
Cytology analysis of pericardial fluid shows malignant cells, which contain enlarged hyperchromatic nuclei with moderate nuclear atypia. The background shows blood (magnification ×400).

She was subsequently transferred to the medical ward, where she continued to have persistent chest discomfort and tightness. Concomitantly, her dry cough and reduced breath sounds in the right lower zone of the lung raised the suspicion of a noncardiac pathology. An echocardiogram was done as part of the routine investigation for ACS, which revealed moderate to massive pericardial effusion and dilated inferior vena cava (24 mm) with less than 50% respiratory variation. She underwent pericardiocentesis, draining approximately 450 mL of hemorrhagic pericardial fluid. The pericardial fluid analysis showed that 80% of the white blood cells were lymphocytes and the presence of poorly differentiated malignant cells (Figure 2). Following the pericardiocentesis, she described improvement in chest discomfort and tightness.

**Figure 3:**
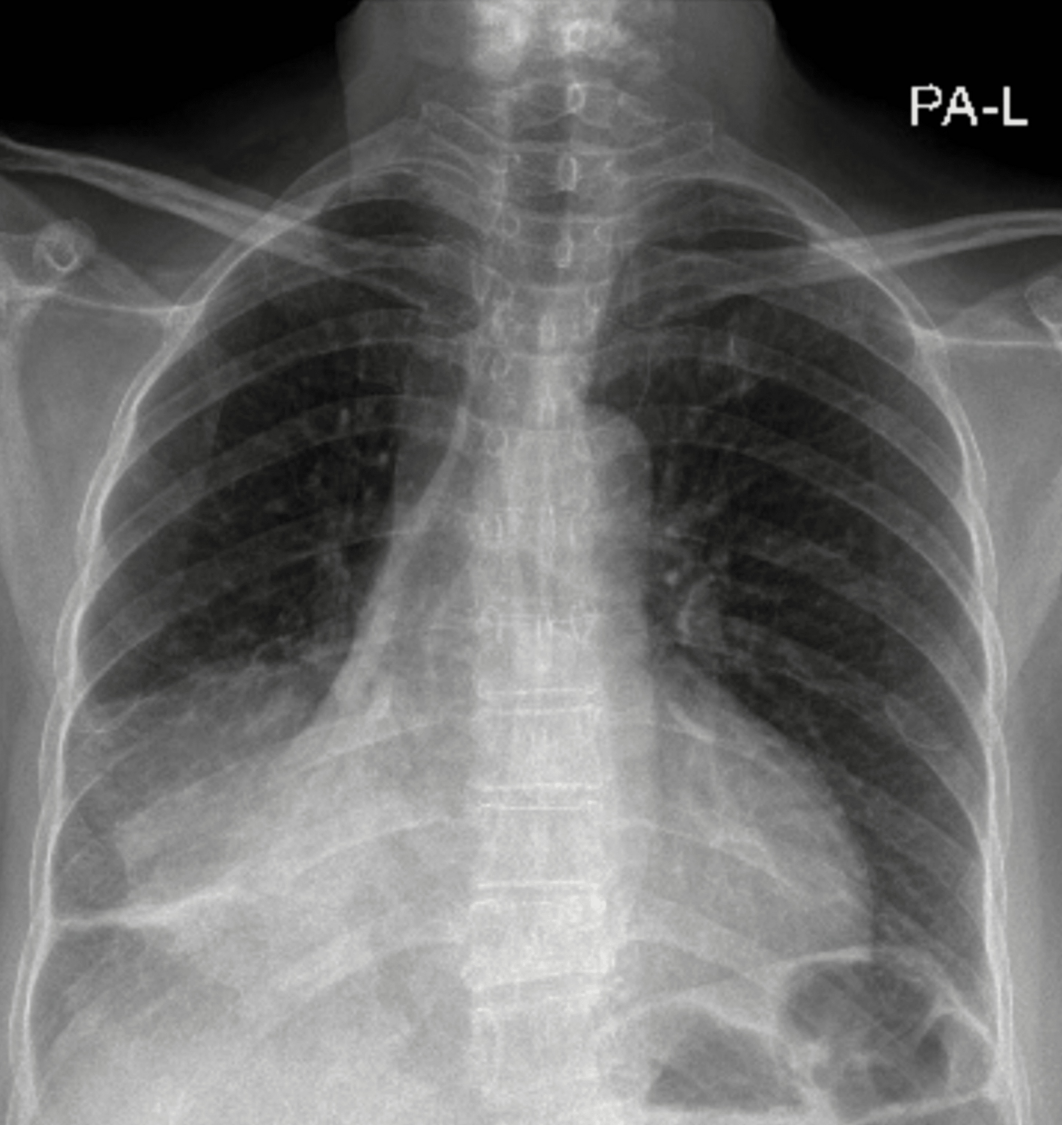
The chest radiography shows a mild increase in cardiac silhouette, tracheal deviation to the right side, and opacification in the right lower zone of the lung, suggestive of a mass lesion with lower lobe collapse.

**Figure 4:**
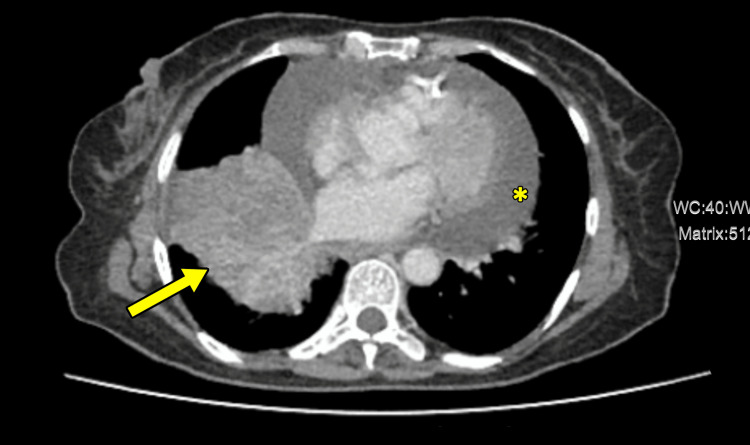
CT chest shows a right lower lobe mass lesion obstructing the lower lobe bronchus, causing collapse of the right lower lobe, suggestive of lung malignancy (arrow). Additionally, there is a significant pericardial effusion (asterisk). CT, computed tomography

Further evaluation for malignant pericardial effusion was done through chest X-ray (Figure 3) and contrast-enhanced computed tomography (CT) of the chest, abdomen, and pelvis. The imaging revealed a mass lesion in the right lower lobe, obstructing the lower lobe bronchus and causing a collapse of the right lower lobe, consistent with lung malignancy (Figure 4). Moreover, the CT scan demonstrated significant pericardial effusion and sclerotic bone metastases.

**Figure 5:**
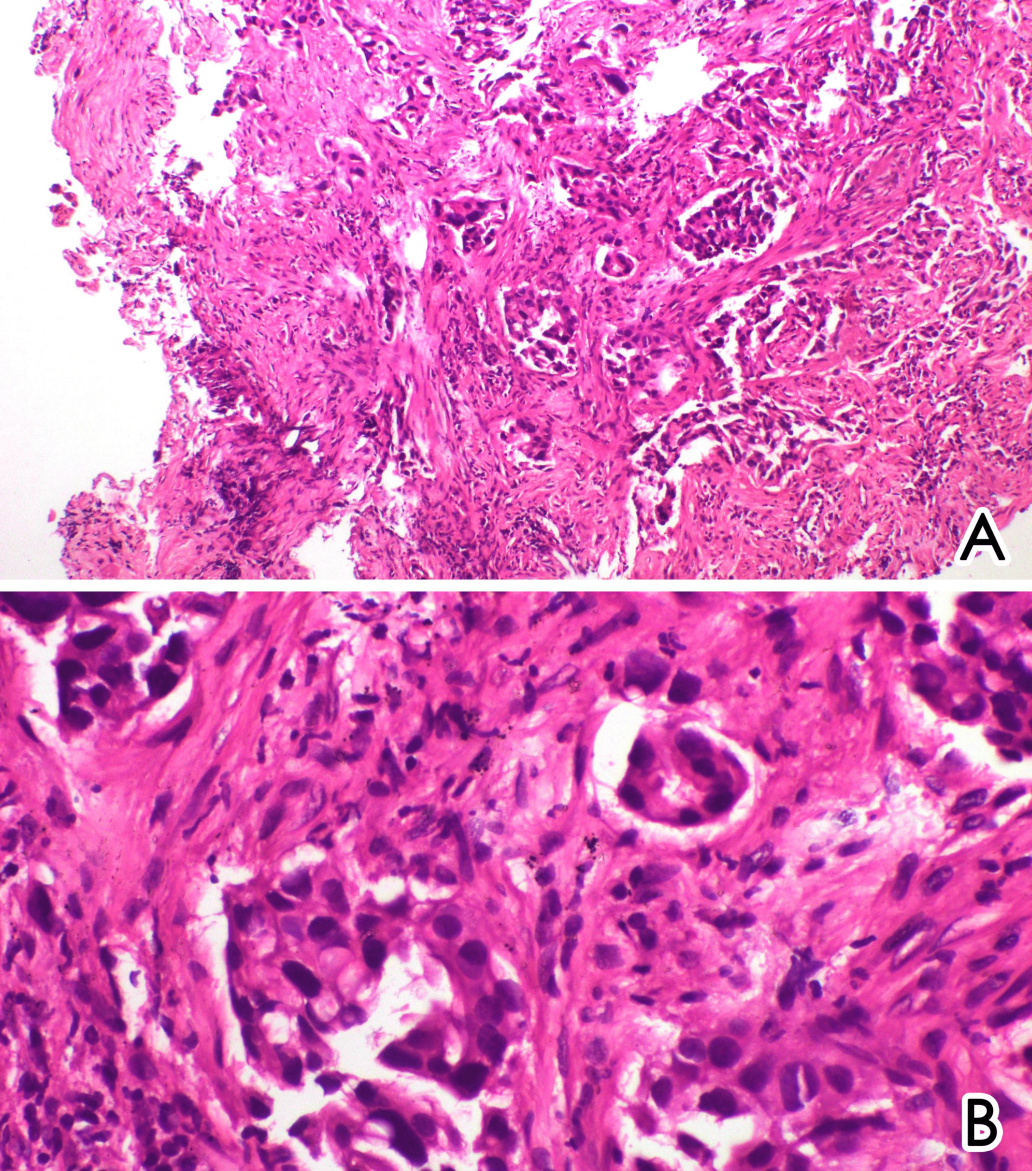
Histology analysis of the endobronchial mass shows a tumor formed of irregular and complex glands in a desmoplastic stroma (A, magnification ×100) lined by columnar epithelial cells containing moderately pleomorphic nuclei with increased mitotic activity (B, magnification ×200).

Subsequently, a bronchoscopy was performed, confirming the presence of a right lower lobe bronchial mass. A biopsy of the endobronchial mass was obtained, and histopathological examination revealed invasive adenocarcinoma of the lung (Figure 5). She was transferred to the oncology department for further management.

## Discussion

This case emphasizes the complexity of the evaluation of chest pain and the importance of keeping a list of differential diagnoses. While ACS is a common and critical cause of chest pain, other potential etiologies must be considered especially when initial measures do not yield the expected results and presence of atypical clinical features. In this case, the presentation with chest pain and exertional dyspnea raised concerns for ACS with the ECG changes and mildly elevated troponin I levels. However, the persistence of symptoms despite appropriate management and associated mild dry cough with reduced breath sound in the right lower zone warranted further evaluation.

The discovery of moderate to massive pericardial effusion on echocardiogram and the cytological findings of malignant cells in the hemorrhagic pericardial fluid were crucial in redirecting the diagnostic focus. Subsequent CT imaging revealed a mass in the right lower lobe, obstructing the bronchus and causing lung collapse, together with sclerotic bone metastases. The biopsy of the mass lesion confirmed the diagnosis of invasive adenocarcinoma of the lung, providing a clear explanation for the patient’s symptoms.

Malignant pericardial effusion due to lung malignancy, particularly adenocarcinoma, is a well-documented but relatively rare cause of chest pain. Lung mass itself also causes chest pain. Several case reports and case series highlight the diagnostic challenges and clinical presentations of such cases. For instance, a study by Ben-Horin et al. described a series of patients with malignant pericardial effusion, emphasizing the different clinical presentations and the essential cytological analysis of pericardial fluid [3]. A case report by Vemireddy et al. described a 57-year-old female who presented with cardiac tamponade and subsequent investigations revealed that the underlying cause was lung adenocarcinoma [4].

Another case report by Dessalegn et al. described a 40-year-old male, a nonsmoker, who presented with epigastric pain accompanied by nausea and vomiting. Subsequent imaging studies revealed a large pericardial effusion with tamponade effect and a left lung upper lobe mass lesion. CT-guided biopsy was done and was consistent with adenocarcinoma of the lung [5]. A case series published in 2014 described cardiac tamponade complicating malignant pericardial effusion from non-small cell lung cancer. The study found that adenocarcinoma was the most common histological subtype associated with pericardial involvement [6]. A study by Adler et al. on the diagnostic approach to pericardial diseases emphasized the role of multimodal imaging (echocardiography, CT, and MRI) and the importance of cytological and histological analysis in identifying malignant causes of pericardial effusion [7].

Pericarditis with effusion, regardless of etiology, can be associated with a spectrum of ECG changes: generalized ST elevation with reciprocal changes in aVR during the first two weeks; normalizing ST changes with generalized T-wave flattening between 1-3 weeks; flattened T waves followed by inverted T waves after three weeks; and eventually, a return to normal ECG. However, less than half of cases show these classical changes in ECG [8]. Our patient had T-wave inversion in multiple ECG leads, which was initially thought to be due to ACS. However, troponin I can be elevated in various conditions, including pericardial diseases, as seen in our case [9].

This case illustrates several key lessons. Clinicians should maintain a broad differential diagnosis for chest pain, particularly when initial treatments for ACS are ineffective and when other clinical features suggest alternative diagnoses. Alternative diagnoses such as pulmonary pathology and pericardial diseases should be considered. Multimodal imaging and cytological analysis have a central role in the diagnostic workup of pericardial effusion as in this case. Managing complex cases like this requires an interdisciplinary approach, involving a cardiologist, respiratory physician, radiologist, and histopathologist to ensure a comprehensive evaluation.

## Conclusions

This case highlights the diagnostic challenges of chest pain and the clinical reasoning required in managing chest pain. It emphasizes the importance of considering a broad differential diagnosis and employing a comprehensive diagnostic workup. This case provides valuable insights for clinicians encountering similar diagnostic dilemmas.
